# Mapping Gene Associations in Human Mitochondria using Clinical Disease Phenotypes

**DOI:** 10.1371/journal.pcbi.1000374

**Published:** 2009-04-24

**Authors:** Curt Scharfe, Henry Horng-Shing Lu, Jutta K. Neuenburg, Edward A. Allen, Guan-Cheng Li, Thomas Klopstock, Tina M. Cowan, Gregory M. Enns, Ronald W. Davis

**Affiliations:** 1 Stanford Genome Technology Center, Stanford University, Palo Alto, California, United States of America; 2 Institute of Statistics, National Chiao Tung University, Hsinchu, Taiwan; 3 BioMarin Pharmaceutical, Novato, California, United States of America; 4 Department of Operations Research, University of California Berkeley, Berkeley, California, United States of America; 5 Friedrich-Baur-Institute, Department of Neurology, Ludwig Maximilians University, Munich, Germany; 6 Departments of Pathology and Pediatrics, Stanford University, Stanford, California, United States of America; 7 Department of Pediatrics, Medical Genetics Division, Stanford University, Stanford, California, United States of America; University of Chicago, United States of America

## Abstract

Nuclear genes encode most mitochondrial proteins, and their mutations cause diverse and debilitating clinical disorders. To date, 1,200 of these mitochondrial genes have been recorded, while no standardized catalog exists of the associated clinical phenotypes. Such a catalog would be useful to develop methods to analyze human phenotypic data, to determine genotype-phenotype relations among many genes and diseases, and to support the clinical diagnosis of mitochondrial disorders. Here we establish a clinical phenotype catalog of 174 mitochondrial disease genes and study associations of diseases and genes. Phenotypic features such as clinical signs and symptoms were manually annotated from full-text medical articles and classified based on the hierarchical MeSH ontology. This classification of phenotypic features of each gene allowed for the comparison of diseases between different genes. In turn, we were then able to measure the phenotypic associations of disease genes for which we calculated a quantitative value that is based on their shared phenotypic features. The results showed that genes sharing more similar phenotypes have a stronger tendency for functional interactions, proving the usefulness of phenotype similarity values in disease gene network analysis. We then constructed a functional network of mitochondrial genes and discovered a higher connectivity for non-disease than for disease genes, and a tendency of disease genes to interact with each other. Utilizing these differences, we propose 168 candidate genes that resemble the characteristic interaction patterns of mitochondrial disease genes. Through their network associations, the candidates are further prioritized for the study of specific disorders such as optic neuropathies and Parkinson disease. Most mitochondrial disease phenotypes involve several clinical categories including neurologic, metabolic, and gastrointestinal disorders, which might indicate the effects of gene defects within the mitochondrial system. The accompanying knowledgebase (http://www.mitophenome.org/) supports the study of clinical diseases and associated genes.

## Author Summary

An important prerequisite for successful disease gene identification is the assessment, with minimal ambiguity, of a particular clinical trait or phenotype. Even with years of experience, recognizing and diagnosing mitochondrial diseases is still a major hurdle in clinical medicine. Computational tools supporting clinicians not only help identify affected individuals, but also guide studies of the genetic and biological causes of these disorders. In this study we dissect and categorize individual clinical features, signs, and symptoms of 174 disease genes and then identify gene similarities based on their shared phenotypic features. We demonstrate that genes sharing more similar phenotypes have a stronger tendency for functional interactions, proving the usefulness of phenotype similarity values in disease gene network analysis. Our study of a large functional network of mitochondrial genes revealed distinct properties that differentiate disease and non-disease genes. Disease genes showed a lower average total connectivity but a tendency to interact with each other; a finding that we used to predict 168 high-probability disease candidates. The accompanying knowledgebase allows for easy navigation between disease and gene information. We believe the open source format will support and encourage further research that will benefit this and other human phenome projects.

## Introduction

Mitochondrial diseases are caused by an abnormal function of mitochondria. They may be the result of spontaneous or inherited mutations in the mitochondrial genome (mtDNA) or in nuclear genes that code for mitochondrial components, but may also be acquired secondary to adverse effects of drugs, infections, or other environmental causes [1]–[3]. The mtDNA encodes only 13 proteins of the respiratory chain [4], while most of the estimated 1,500 mitochondrial proteins are nuclear-encoded [5]. Mitochondrial deficiencies often affect multiple tissues leading to multi-system diseases that present with many phenotypic features. These dysfunctions appear to be more prevalent in hereditary diseases than previously anticipated [6]–[8] and have also been attributed to the pathogenesis of common conditions associated with aging [3],[9] including neurodegenerative diseases [10], cardiovascular disorders [11], diabetes mellitus [12], and several cancer types [13],[14].

Medical case reports of specific gene defects have been crucial to our understanding of clinical phenotypes. The list of mitochondrial disease genes and case reports has grown rapidly, while methods for defining and assaying clinical phenotypes are still inadequate [15]–[17]. Accordingly, the accurate and systematic comparison of clinical phenotypes associated with different disease genes remains a major challenge. One limitation is the non-standardized formats of such phenotypic data in the medical literature and databases, which is difficult to overcome using automated text mining [18],[19]. An example are optic nerve diseases for which multiple terms are found such as cranial nerve II diseases, neural-optic lesion, optic disk disorder, and optic atrophy. Higher-level phenome knowledgebases recently emerged in an attempt to comprehensively index human phenotype data [20]–[22]. The process of transforming descriptions of medical diagnoses and procedures into universal computer-readable medical code numbers involves manual reviews and annotations of full-text articles [17]. As with other knowledgebases [23],[24], catalogs of clinical phenotypes are set within the context of the existing literature, but are also limited by the inherent problems of working with an evolving literature.

In this study, we catalogued detailed information on clinical disease phenotypes of known mitochondrial gene defects that were stored in a phenome knowledgebase. We then developed methods to analyze the clinical phenotype information, to determine associations of genes and diseases, and to compare different disease genes based on their associated phenotypes. This approach was used to predict disease gene similarities, which showed positive correlations to their functional interactions. Our analysis of a functional interaction network of mitochondrial genes revealed distinct properties for disease and non-disease genes, which we utilized to predict new disease candidate genes. Our knowledgebase (www.mitophenome.org) represents a new resource for studying links between disorders and genes. This can be integrated with a variety of systems approaches [25] with the goal of identifying disease gene variants in the individuals that carry them.

## Results

### Annotation of mitochondrial genes and diseases

We identified 174 nuclear-encoded mitochondrial genes (**Table S1**) associated with 191 diseases in the Online Mendelian Inheritance in Man (OMIM) database [26]. In order to characterize these disorders in detail, we manually searched the PubMed literature for their phenotypic features such as clinical signs and symptoms, biochemical and clinical laboratory tests, and neurological imaging findings. Our annotations consisted of three steps that included the collection, definition, and classification of phenotypic features for each disease gene. Importantly, we individually matched the phenotypic features with standardized descriptors in the Medical Subject Headings (MeSH) database [27]. Within the hierarchical MeSH ontology, we localized the individual feature position and then identified, for each feature, the directly related parent descriptor or hypernym feature. Using this approach, we reviewed 1,636 full-text articles reporting defects or deficiencies in the 174 disease genes and individually extracted phenotypic features for each gene. We then matched features with MeSH descriptors and identified their hypernyms, which generated 502 features hierarchically classified within the mitochondrial phenotype ontology (**Table S2**). At its root, the ontology has fourteen features corresponding to fourteen major clinical categories (CC) such as cardiovascular diseases or neurological diseases. These CC are used to discriminate the more specific features in each group: for example arrhythmia in the cardiovascular CC and seizures in the neurologic CC. A subset of the features in our phenotype ontology is listed in **Table 1**.

**Table 1:** Phenotypic features of human mitochondrial diseases.

**1. Cardiovascular** (110)	**7. Immunologic** (78)	Neuromuscular-manifestations (129)
Arrhythmia (38)	Autoimmune-diseases (2)	Paralysis-Paresis (39)
Cardiomyopathy (44)	Immune-deficiency (7)	Reflexes-abnormal (78)
Cardiorespiratory-arrest (81)	Infections (76)	Hearing-disorders (30)
Hypertension (20)	**8. Metabolic** (143)	Voice-disorders (11)
Hypotension (19)	Acidosis (87)	Stroke-like-episodes (10)
Myocardial-ischemia (6)	Reye-like-symptoms (15)	Developmental-delay (111)
**2. Dermatologic** (56)	Dyslipidemias (14)	Polyneuropathies (37)
Dermatitis (7)	Diabetes-mellitus (18)	Sleep-disorders (11)
Hair-diseases (15)	Hyperglycemia (12)	**11. Oncologic** (29)
Pigmentation-disorders (11)	Hyperinsulinism (5)	Squamous-cell-neoplasms (4)
Hyperhidrosis (15)	Hypoglycemia (52)	Neuroendocrine-tumors (6)
Paleness (22)	Hyperammonemia (40)	Paraganglioma (3)
**3. Endocrinologic** (40)	Hyperbilirubinemia (23)	Leukemia-Lymphoma (9)
Adrenal-gland-diseases (14)	Hemochromatosis (11)	Breast-neoplasms (5)
Adrenal-insufficiency (9)	Aminoacid-levels-abnormal (47)	Colorectal-neoplasms (4)
Adrenocortical-hyperfunction (7)	Water-electrolyte-imbalance (49)	Hepatocellular-carcinoma (7)
Gonadal-disorders (23)	Obesity (8)	Leiomyoma (4)
Sex-differentiation-disorders (12)	Fatty-acids-abnormal (21)	Prostatic-neoplasms (4)
Parathyroid-diseases (5)	Organic-acids-abnormal (69)	Renal-cell-carcinoma (4)
Pituitary-diseases (10)	Dicarboxylic-aciduria (20)	**12. Ophthalmologic** (87)
Thyroid-diseases (18)	**9. Musculosceletal** (95)	Blepharoptosis (20)
**4. Gastrointestinal** (132)	Osteoporosis (8)	Cataract (15)
Cholestasis (16)	Spinal-diseases (25)	Pathologic-nystagmus (38)
Deglutition-disorders (29)	Pathological-fractures (9)	Ophthalmoplegia (15)
Gastroenteritis (28)	Foot-deformities (20)	Optic-nerve-diseases (34)
Intestinal-obstruction (13)	Joint-diseases (18)	Retinal-diseases (27)
Gastrointestinal-hemorrhage (9)	Muscular-diseases (38)	Color-vision-defects (7)
Liver-diseases (79)	Rhabdomyolysis (8)	**13. Psychiatric** (51)
Fatty-liver (34)	Microcephaly (39)	Aggression (15)
Pancreatitis (9)	**10. Neurologic** (154)	Feeding-behavior (8)
Abdominal-pain (31)	Brain-diseases (135)	Anxiety-disorders (18)
Feeding-difficulties (67)	Intracranial-hemorrhages (17)	Dementia (18)
Diarrhea (37)	Seizures (97)	Autistic-disorder (8)
Vomiting (80)	Headache-disorders (21)	Depressive-disorder (19)
**5. Genitourinary** (68)	Leukoencephalopathy (39)	Psychotic-disorders (20)
Infertility-male (7)	Cerebellar-atrophy (28)	Schizophrenia (8)
Hypospadias (6)	Corpus-callosum-hypoplasia (18)	**14. Respiratory** (108)
Cystic-kidney-diseases (7)	Choreatic-disorders (18)	Hyperventilation (40)
Nephrocalcinosis (4)	Dystonic-disorders (38)	Respiratory-insufficiency (51)
Renal-insufficiency (30)	Parkinsonian-disorders (12)	Asthma (8)
Urination-disorders (12)	Tremor (36)	Pneumonia (40)
Menstruation-disturbances (9)	Spinal-cord-diseases (29)	Pulmonary-edema (14)
Pregnancy-complications (15)	Neurogenic-bladder (10)	**Miscellaneous** (151)
**6. Hematologic** (75)	Ataxia (54)	Fever (52)
Anemia (34)	Speech-disorders (41)	Hypothermia (17)
Blood-coagulation-disorders (24)	Consciousness-disorders (65)	Exercise-intolerance (36)
Petechiae (4)	Memory-disorders (13)	Failure-to-thrive (62)
Blood-platelet-disorders (21)	Mental-retardation (68)	Growth-deficiency (54)
Blood-protein-disorders (20)	Hallucinations (14)	Dysmorphisms-abnormalities (39)
Bone-marrow-diseases (20)	Psychomotor-agitation (34)	Odors (10)
Leukocyte-disorders (25)	Irritability (39)	RCC-deficiencies (42)
Lymphatic-diseases (8)	Lethargy (69)	Vitamin-responsive (24)

The 144 features are selected from a total of 502 features (**Table S2**) and are caused by defects in 174 nuclear-encoded mitochondrial genes. Every feature is associated with the number of genes shown in parentheses. The hierarchical structure of features within the phenotype ontology was established using standardized MeSH descriptors (not shown). The fourteen CC in bold serve as headers for features within them. Unassigned features are grouped under ‘Miscellaneous’.

### Clinical categories (CC) of mitochondrial disorders

**Figure 1:**
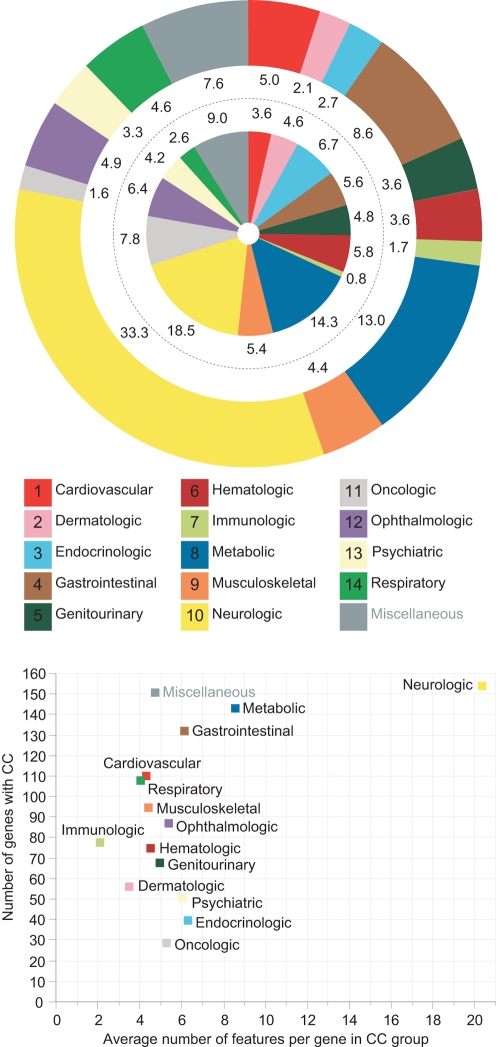
Distribution of clinical phenotypic features in mitochondrial diseases. (A) The inner circle shows the distribution of 502 phenotypic features among fourteen clinical categories (CC), plus a ‘Miscellaneous’ category containing unassigned features. The numbers show the fraction in % of all features in one CC compared to all 502 features. The outer circle shows the distribution of features related to CC within the 9,407 gene-feature pairs, with the frequency in % of all features in one CC. (B) Number of genes with features in a specific CC (y-axis) in correlation to the average number of CC-specific features caused by these genes (x-axis). 154 genes caused neurological features with an average of 20.2 neurological features per gene. Phenotypically, most mitochondrial gene defects are related to neurological, metabolic and gastrointestinal diseases.

A categorical breakdown of the 502 features in their fourteen CC is shown in the inner circle of **Figure 1A**. While most CC were comprised of more than twenty individual features, the neurologic and metabolic CC contained the largest fraction of features (18.5% and 14.3%, respectively). We then explored the overall characteristics of mitochondrial phenotypes across all gene defects. We had annotated a total of 9,407 gene-feature pairs (**Table S3**) that included, for each of the 174 disease genes, features identified through our literature search and hypernyms to these features assigned through integration with the phenotype ontology (see methods). A relative breakdown of the fourteen CC across the 9,407 gene-feature pairs is shown in the outer circle of **Figure 1A**. This analysis revealed CC patterns similar to the categorical distribution above, with neurological (33.3%) and metabolic (13.0%) features most prominently represented. Together with the third largest CC of gastrointestinal (8.6%) diseases, these three CC account for more than half of all features in all gene-feature pairs studied. In comparison, the oncologic and endocrinologic CC contained relative large numbers of features, but these categories were associated with fewer genes and are less frequently observed in mitochondrial disorders.

The distribution of phenotypes within CC is largely consistent with the tissue distribution of energy expenditure in the resting state, or basal metabolic rate (BMR), with brain contributing to the highest proportion of the BMR (90% in newborns, 60% in infants, and 25% in adults) [28], followed by liver (20–25% BMR) and resting muscle (10–25% BMR). Mitochondria provide most of the body's energy [3], and measurements of mitochondrial respiration have shown that brain tissue contains more active respiratory chain complexes than liver, heart, or muscle [29]. Thus, our results showing a higher proportion of neurological, metabolic, and gastrointestinal (e.g. liver diseases) features positively correlate to BMR and respiratory-chain activities. A related analysis of genes associated with the fourteen CC confirms this observation (**Figure 1B**). While most genes were associated with the neurologic, metabolic or gastrointestinal CC, these genes also caused more features within these CC. For example, 154 genes were associated with the neurological CC with each gene causing on average 20.3 neurological features. Although mitochondrial defects affect many cellular processes [5], the phenotype patterns predominantly represent deficiencies in energy metabolism with the nervous system being most susceptible. Like the gene-expression patterns in the study of human phenotypic diversity [30], CC patterns may aid to characterize and distinguish phenotype groups such as mitochondrial disorders.

### Clinical phenotype similarities between mitochondrial disease genes

Inherited diseases often present with multiple phenotypic features. The presence or absence of specific features is traditionally used to distinguish between different disorders and identify clinical traits [26]. It is hypothesized that phenotype similarities of different disorders may indicate biological relationships of the underlying genes [15],[16]. Several systematic studies have recently investigated genotype-phenotype associations in human genetic disorders [19],[31],[32]. These approaches utilized automated text mining to extract phenotype information, while noting that currently available text formats and databases were not designed as structured resources for human phenotype analysis [19]. Another study utilized disorder terms from OMIM's Morbid map and manually annotated each disorder into one of 22 disorder classes [33], which are comparable to our CC. Notably, the Morbid map terms represent only a small fraction of the phenotype information of a clinical disorder and genes with identical terms may cause phenotypic features from different CC. In order to explore associations of genes and features in mitochondrial diseases, we utilized our manually annotated 9,407 gene-feature pairs (**Table S3**). Evidence for each gene-feature association is derived from one or more of 1,636 full-text articles, where each article is linked to a PubMed unique identifier (PMID). Using the PMID we computed the association ratio for each gene-feature pair. This ratio represents the number of PMID reporting a specific feature for a specific gene out of all PMID annotated for this gene. In addition, we determined the association ratios of pairs of genes linked to the same feature (see Methods). The integration of association ratios for all features related to a gene pair enabled the prediction of quantitative phenotypic associations (QPA). Thus, QPA are a quantitative measure of phenotype similarity of disease genes causing one or more identical phenotypic feature.

### Correlation of phenotypic associations and gene functional interactions

We then compared disease genes with QPA and functional interactions, in order to explore the hypothesis of phenotypic similarities in functionally related genes [15],[16]. We identified 1,928 gene pairs (n = 139 genes) from a recent study with Likelihood Ratios (LR) for gene functional interactions [34], and for which we had predicted QPA (**Table S4**). Using rank correlation, we detected positive associations with significant confidence of QPA and LR for these disease gene pairs (Kendall: p = 4.67e-7; Spearman: p = 3.72e-7). The results indicated that genes with stronger evidence for functional interaction (higher LR) displayed greater similarities in their associated disease phenotypes (higher QPA). To select gene pairs with the highest correlation of LR and QPA, we applied hierarchical clustering and identified groups of gene pairs with higher to lower levels of association (see Methods). In addition, we compared the 1,928 gene pairs with both LR and QPA to pairs predicted by only one method. Hypothesis testing revealed that these pairs showed on average higher values for LR (p = 6.86e-10) and QPA (p = 0.029) than pairs predicted by only one method (LR pairs only n = 82; QPA pairs only n = 26,010). Thus, LR and QPA in combination could be helpful in the analysis of disease gene associations.

**Figure 2:**
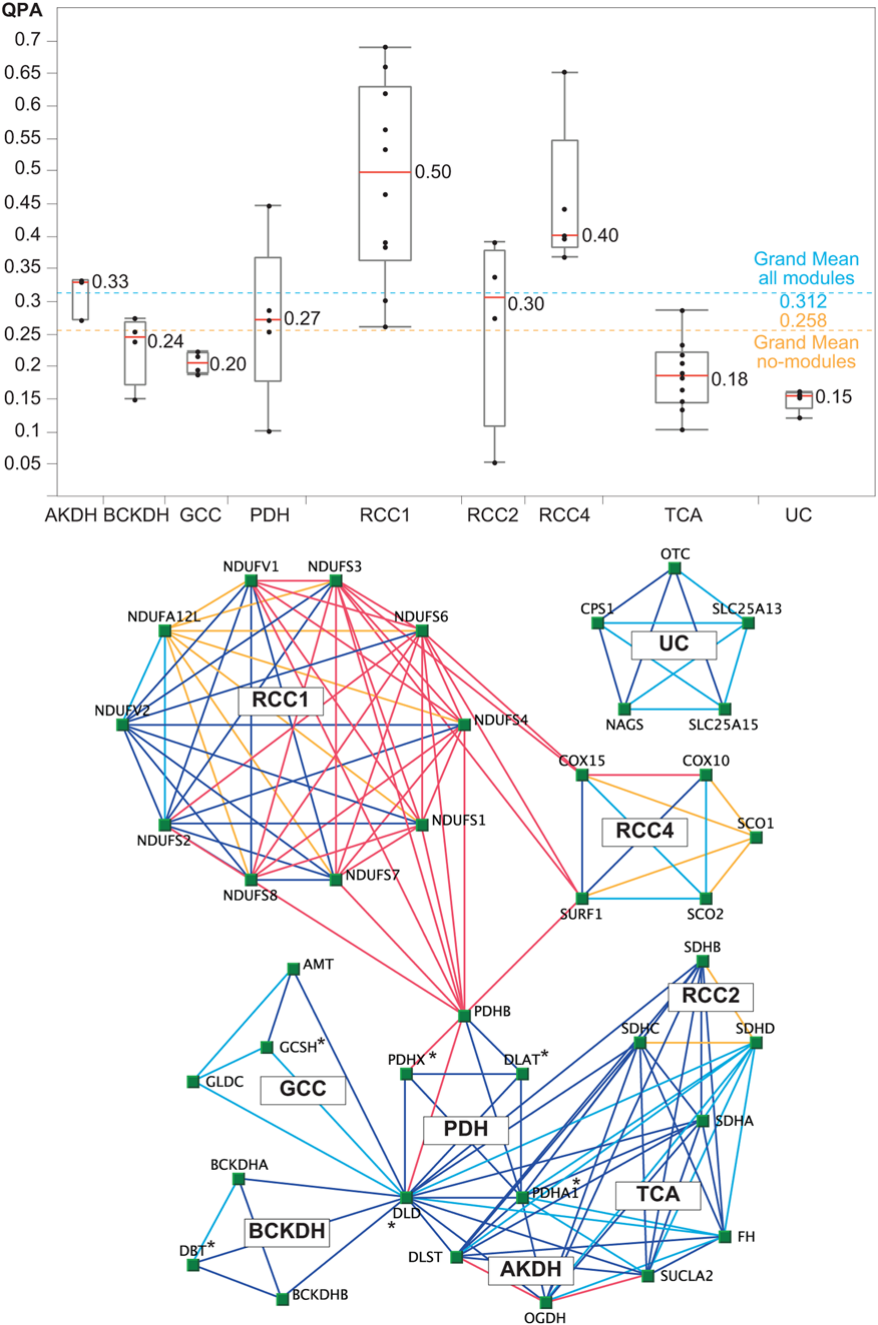
Phenotype similarity of genes related to mitochondrial protein complexes and pathways. (A) For each gene (black dots), the average QPA (y-axis) to all other genes within a functional module was calculated. Red lines represent the median (50th percentile) of all QPA averages within a module. Boxes indicate the 25th and 75th quartiles, with minimum and maximum data points as lines that extend from each end of the box. The grand mean of all modules (blue line) is the QPA average across all gene pairs of all nine modules, which was significantly higher than for pairs outside modules (orange line). (B) Module gene relationships are predicted through functional (LR) and phenotypic (QPA) associations showing the usefulness of phenotype similarity scores in disease gene network analysis. The edge colors are: Red – gene pairs with highest correlation of QPA and LR; blue – gene pairs with lower QPA-LR correlation; orange and light blue – gene pairs with QPA only at higher (> = 0.4) and lower confidence (<0.4), respectively (see Table S4 for data). Diseases caused by the six genes labeled “*” are known to respond to vitamin treatments (riboflavin, thiamine, and pyridoxine). Abbreviations: AKDH, Alpha ketoglutarate dehydrogenase; BCKDH, Branched chain alpha keto acid dehydrogenase; GCC, Glycine cleavage system; PDH, Pyruvate dehydrogenase; RCC, Respiratory chain complex; TCA, Tricarboxylic acid cycle; UC, Urea cycle.

In our next analysis we identified 39 disease genes encoding components of seven mitochondrial protein complexes and two metabolic pathways, representing nine functional modules (**Table S4**). For each gene within a given module, we calculated the QPA average relative to all other genes in the module (**Figure 2A**). While genes within some modules were associated with similar disease phenotypes (e.g. RCC1, RCC4), other modules appeared phenotypically more diverse (e.g. BCKDH, TCA). We then compared QPA of gene pairs within modules (n = 262 pairs) to pairs outside modules (n = 27,676 pairs). This analysis revealed a higher average phenotype similarity for gene pairs within versus outside the nine modules (p = 2.64e-5). We found a comparable result in the analysis of gene functional interactions, with on average higher LR for gene pairs within (n = 182) versus pairs outside these modules (n = 1,828; p = 1.86e-35). In summary, we identified positive correlations of functional (LR) and phenotypic (QPA) associations for many disease genes, with the most prominent genotype-phenotype relationships in protein complexes (**Figure 2B** **)**. These results support findings of a recent study that utilized automated text mining in OMIM to identify phenotypic similarities within protein complexes [32]. However, it should be noted that OMIM often combines genes into a single disease record, if they encode subunits of the same protein complex (e.g. BCKDH - Maple syrup urine disease, #248600; GCC - Glycine encephalopathy, #605899). Potential circular reasoning in correlating phenotypes and complexes could be reduced by individual disease gene annotations. While statistically very significant, the genotype-phenotype correlation values observed in this and other studies are still rather small [35]. Possible contributing factors are the imperfect information about gene-gene and gene-disease associations and the environment.

### Functional interactions of nuclear-encoded mitochondrial genes

We then expanded our analysis and identified functional interactions for 162 disease genes (DG) to 4,577 candidate genes (CG) from a recent study [34]. As for the DG, we also extracted all binary functional interactions for each CG in order to account for all genome-wide interactions of all 4,739 genes studied (**Table S5**). Of the CG, 531 genes had disease associations in OMIM [26] and we consequently labeled those as DG. We recorded in total more than 1.9 million gene interactions that included interactions between disease genes (DG-DG), disease and candidate genes (DG-CG, CG-DG), and candidate genes (CG-CG). We first focused on the mitochondrial gene network and identified a set of 495 mitochondrial CG through data integration of two recent studies [5],[36]. These studies combined had predicted 1,200 human mitochondrial genes (**Table S6**). Our analysis of functional interactions of all mitochondrial CG (495) and DG (162) showed the following results: i. the total number of interactions (i[all-genes]), which is recorded to all human genes, was higher for CG than for DG (p = 7.93e-6); ii. the relative number of DG interactions (i[disease-genes]/i[all-genes]), which was recorded to all known human DG, was higher for DG than for CG (p = 6.37e-7); and iii. the relative number of interactions to human orthologs of mouse (i[mouse-essential]/i[all-genes]) and yeast (i[yeast-essential]/i[all-genes]) essential genes [33],[37] was higher for the DG than the CG (mouse p = 1.72e-4; yeast p = 0.013). These results indicated that mitochondrial CG and DG can be distinguished based on functional interaction patterns.

**Figure 3:**
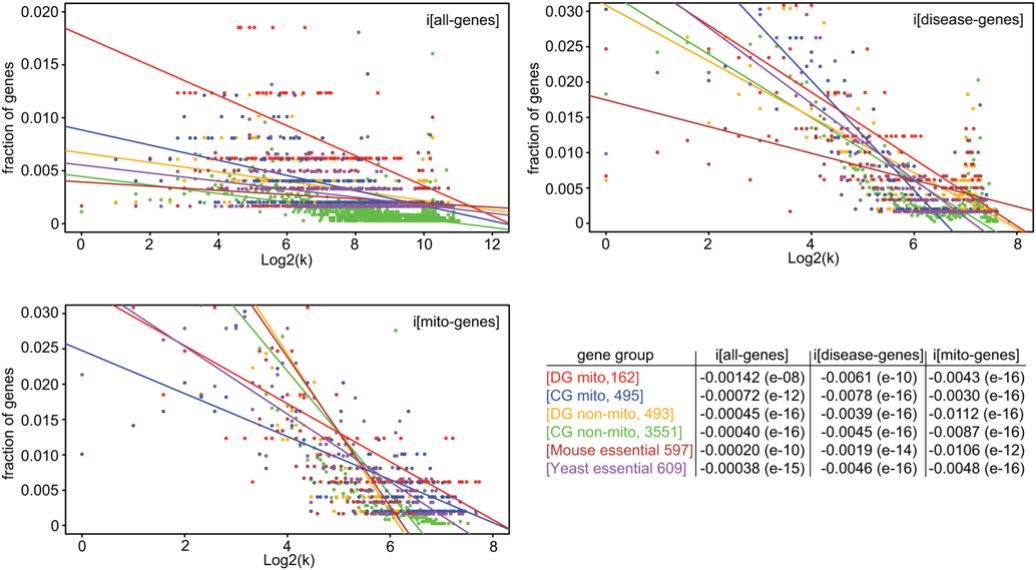
Functional interactions of human mitochondrial genes. For each gene group, we calculated the fraction of genes (y-axis) that interact with k other genes (x-axis). The gene group fractions were calculated for interactions to all human genes (A), all disease genes (B), and all mitochondrial genes (C). The color codes of the gene groups with their respective regression line slopes and p-values (in parenthesis) are shown in the table (D), with all results including correlation coefficients listed in Table S7. The fraction analysis of all gene interactions (A) showed a higher network connectivity for non-disease genes (CG) than for disease genes (DG), as indicated by the larger absolute value of the negative DG regression slope. In contrast, the connectivity to disease genes is relatively higher for DG than for CG suggesting a tendency for DG to interact with each other (B). Human orthologs to mouse essential genes had the highest network connectivity, while the mitochondrial gene groups had the highest tendency to interact to other mitochondrial genes (C).

In a related analysis, we compared the functional interactions of DG (493) and CG (3551) located outside the mitochondrial organelle (**Table 2**), which showed similar results (i., ii. and iii.) as for the mitochondrial gene groups (see Methods for data). However, the comparison of mitochondrial and non-mitochondrial genes revealed some surprises. The total number of interactions for non-mitochondrial DG was higher than for mitochondrial DG (p = 6.5e-29), and the number of interactions for non-mitochondrial CG was higher than for mitochondrial CG (p = 2.37e-93). Notably, the non-mitochondrial DG had on average more interactions than the mitochondrial CG (p = 1.87e-19). To further investigate these differences, we literature-annotated detailed information on intracellular localizations of gene products (**Table S1**). Out of the 162 DG, 115 genes had only evidence for mitochondrial localizations, while 47 DG also localized to additional compartments (e.g. nucleus, cytoplasm). In addition, we identified 38 DG out of the 4,577 CG (**Table S6**) with likely mitochondrial localizations [5],[36]. These 38 DG (p = 0.06) and the 47 DG with multiple localizations (p = 0.51) tended to have more interactions than the 115 mitochondria-only DG although both results were not statistically significant. In summary, our analysis identified a higher average connectivity for non-disease genes (CG) than for DG, which was detected for both mitochondrial and non-mitochondrial genes, and secondly, fewer functional interactions of mitochondrial than for non-mitochondrial genes. These findings are supported by a separate gene fraction analysis (**Figure 3**), where we studied the number of interactions of genes in the different gene groups and the distribution of these interactions over the whole network (see Methods).

**Table 2:** Molecular interactions of mitochondria and non-mitochondria genes.

Gene group (genes/group)	i[all-genes]	i[disease-genes]	i[mouse-essential]	i[yeast-essential]	i[mito-genes]
DG mitochondria (162)	120.91 (62)	26.14 (17.5)	13.23 (6)	20.56 (8.5)	42.24 (22)
CG mitochondria (495)	190.19 (114)	23.93 (14)	14.75 (6)	36.51 (23)	49.81(37)
DG non-mitochondria (493)	396.71 (154)	48.29 (22)	75.01 (17)	29.86 (19)	16.91 (8)
CG non-mitochondria (3551)	459.74 (275)	48.81 (21)	78.4 (21)	44.48 (34)	21.38 (10)
DG mito-only local. (115)	114.48 (67)	27.23 (19)	10.68 (6)	20.14 (9)	48.07 (25)
DG mito+other local. (47)	136.66 (56)	23.47 (14)	19.47 (7)	21.57 (6)	27.98 (18)
DG mito-predict local. (38)	192 (107)	31.18 (18)	22.03 (9)	36.13 (15)	45.76 (30)
Mouse essential genes (597)	598.22 (480)	69.64 (52)	116.7 (63)	39.94 (30)	17.31 (8)
Yeast essential genes (609)	354.96 (230)	33.51 (18)	40.51 (16)	66.15 (54)	37.03 (25)

The total 4,739 genes studied are separated into nine gene groups with the number of disease genes (DG) and candidate genes (CG) in each group in parenthesis (see **Table S5** for individual gene data). DG products with intracellular localization to only mitochondria (115 genes) and mitochondria-and-other-localizations (47 genes) are subsets of the 162 mitochondrial DG. The human orthologs to mouse and yeast essential genes are subsets of all 4,739 genes. The five data columns show the average number of interactions (i) of each group to all genes in the human genome (all-genes); all known human disease genes (disease-genes); all human orthologs of essential mouse genes (mouse-essential); all human orthologs of essential yeast genes; and to all nuclear-encoded human mitochondrial genes (mito-genes). The numbers in parenthesis show the median number of interactions for each group and attribute, respectively. The findings indicated distinct properties in gene molecular interactions for DG and CG, as well as for mitochondria and non-mitochondria genes.

### Candidate genes for mitochondrial disorders

In the last decade, several systematic studies have predicted functional candidate genes in genomic linkage intervals of mitochondrial diseases [38]–[42]. In principle, all genes from a given interval are “benchmarked” against a database of annotated proteins [5], and genes identical to or functionally similar to the reference proteins are prioritized for mutational screens in affected individuals. Here, we build on the success of these approaches and predict new DG from a larger list of mitochondrial CG. Considering the identified interaction differences of disease and non-disease genes, we performed a supervised discriminant analysis [43] of all 695 mitochondrial genes using the five attributes of gene functional interactions (**Table S5**). Out of the 495 mitochondrial CG, 254 genes were predicted as DG with a true positive rate of 80.2% based on the confirmed known DG. In addition, 26 of the 38 DG with likely mitochondrial localization, which we input-labeled as CG to serve as controls, were correctly classified as DG. As an alternative tool, we ran a supervised Bayesian network approach [44],[45]. We first defined a training set of 100 typical out of the 162 mitochondrial DG based on their median of total gene interactions. Accordingly, 100 typical CG were selected from the 495 mitochondrial CG. The network analysis correctly identified 56.8% of the DG, 16 out of the 38 likely mitochondrial DG, and predicted 201 DG out of the 495 CG. Overlapping the two approaches predicted 168 novel mitochondrial DG with an estimated true positive rate of 85.8% (139 out of 162 DG) based on the correctly classified DG (**Table S8**). The newly predicted disease candidates can be prioritized from the larger set of functional CG in linkage intervals of mitochondrial disorders (**Table 3**).

**Table 3:** Prioritizing candidate genes for mitochondrial disorders.

Mitochondrial Disorder	OMIM	Phenotypic features	Linkage interval	Size (Mb)	Gene loci	Mitochondrial genes marked (*) if known, and (^#^) if predicted disease gene
Optic atrophy 2, OPA2	311050	optic nerve disease	DXS993-DXS991	14.5	352	MAOA*, ALAS2*, HSD17B10*; MAOB^#^, **NDUFB11^#^**, **TIMM17B^#^**; ARAF
Optic atrophy 4, OPA4	605293	optic nerve disease	D18S34-D18S479	8.8	58	ATP5A1^#^, **ACAA2^#^**
Optic atrophy 5, OPA5	610708	optic nerve disease	D22S1148-D22S283	10.4	189	HSCB^#^, PISD^#^, **TXN2^#^**, **UCRC^#^**, **TST^#^**; NIPSNAP1, MTP18
Optic atrophy 6, OPA6	258500	optic nerve disease	D8S1702-D8S1794	12.3	86	UQCRB*, DECR1* PPM2C*; SLC7A13^#^; SLC7A13, FAM82B, MTERFD1
Thyroid carcinoma, nonmedullary, TCO	601992	thyroid neoplasms	D19S884-D19S221	4.5	153	TIMM44^#^, **NDUFA7^#^**; MRPL4, FDX1L, ECSIT
Paragangliomas 2, PGL2	601650	neuroendocrine tumors	D11S956-PYGM	6.0	193	BAD^#^, PRDX5^#^, GLYAT^#^, **C11orf79^#^**, **COX8A^#^**; MRPL16, GLYATL1, GLYATL2
Multiple mitochon-drial dysfunction syndrome, MMDFS	605711	muscle weakness, seizures, lethargy, feeding difficulties,	D2S1337-D2S441	8.8	79	**MDH1^#^**; CCT4, ENSG00000119838
Cowchock syndrome; NADMR	310490	muscle weakness, mental retardation, hearing disorder, polyneuropathy	DXS425-HPRT	13.7	152	NDUFA1*; **AIFM1^#^, GLUD2^#^, SLC25A14^#^**
MEHMO syndrome	300148	mental retardation, seizures, obesity, hypogonadism	DXS365-CYBB	15.9	139	GK*; ACOT9^#^, PDK3^#^; APOO
Gustavson syndrome, GUST	309555	mental retardation, optic nerve disease, seizures, deafness	DXS458-DXS424	20.2	285	ACSL4*, TIMM8A*; MCART6, SLC25A5, SLC25A43
Spastic paraplegia, SPG9	601162	paralysis-paresis, cataract, vomiting, foot deformities	D10S564-D10S603	9.4	166	COX15*, ALDH18A1*; NDUFB8^#^, GOT1^#^; C10orf65, SLC25A28

For each mitochondrial disorder (col.1), we identified the mitochondrial candidate genes (col.7) among all gene loci (col.6) in the genomic linkage interval (col.4). The mitochondrial genes are further sorted into: (*) known disease genes with genes (in bold) causing phenotypic features (col.3) similar to features linked to the disease interval; and (^#^) predicted disease genes with genes (in bold) that interact to known disease genes causing features similar to the disease interval features. For completeness, the unlabeled mitochondrial genes are not known or predicted disease genes.

## Discussion

The creation of human phenomic databases has been suggested to systematically collect and analyze phenotypic information [15], [20]–[22]. In this study, we established a clinical phenotype catalog of 174 mitochondrial disease genes (**Table 1**) that account for ∼10% of all known disease genes [26]. In order to define and classify clinical phenotypes from 1,636 medical case reports, we developed a terminologic system that is based on the hierarchical MeSH ontology. Because automated text mining is limited in annotating clinical disorders from the literature [18],[19], our mapping of “phenotypes to language” required the manual review of each full-text article [17]. This classification of phenotypic features for each gene allowed the comparison of disorders between different disease genes (**Figure 1**). To measure clinical phenotype similarity between disease genes, we calculated a numerical value (QPA, quantitative phenotypic associations) that takes into account all annotated gene-feature associations, the overlap of features between two disease genes, and the frequency of the shared feature across all genes. Thus, QPA are based on the hypothesis that the value of a feature varies inversely with the number of genes with which it is associated [16].

The analysis of disease gene pairs with QPA in comparison to Likelihood Ratios (LR) for functional interactions [34] showed positive correlations. Disease genes with stronger evidence for functional interactions (higher LR) displayed greater similarities in their clinical phenotypes (higher QPA). We discovered the most prominent phenotypic similarities within mitochondrial protein complexes (**Figure 2**) supporting previously predicted genotype-phenotype associations of protein complexes [32]. However, we also noted complexes with lower phenotypic similarities (e.g. BCKDH - Maple syrup urine disease; GCC - Glycine encephalopathy) highlighting the importance for individual gene inspection. Since this analysis was limited to disease genes (DG), we were interested in learning the properties of a larger network that included non-disease candidate genes (CG). Utilizing the genome-wide study by Franke et al. [34], we created a functional network of more than 1.9 million gene interactions for 162 mitochondrial DG and 4,577 CG. Our analysis identified significant differences in functional interactions for DG and CG with a higher average connectivity for CG. This difference was detected for both the mitochondrial and non-mitochondrial gene groups (**Table 2**). In addition, while the total number of DG interactions was similar for DG and CG, the relative fraction of DG interactions (i[disease-genes]/i[all-genes]) was higher for DG indicating that DG are more likely to interact with each other. Previous smaller scale studies (∼100× fewer interactions) have predicted intermediate and peripheral positions of DG in gene functional networks with relatively fewer interactions than essential genes [33],[46]. Our results expand on this hypothesis showing that essential and non-disease genes (CG) can be distinguished from DG based on gene interaction patterns (**Figure 3**). Furthermore, we also identified network properties differentiating mitochondrial from non-mitochondrial genes. Mitochondrial genes showed a lower average connectivity, which may be due to the double-membrane structure of the organelle limiting the detection of protein-protein interactions [47]. However, the higher connectivity between mitochondrial genes may relativize this problem. Future studies will help to answer the question of the connectivity of mitochondrial genes and perhaps genes of other cellular compartments as well.

In the final part of this study we utilized the discovered interaction patterns to predict new mitochondrial DG. Using two different approaches, we identified 168 non-disease genes that resembled the characteristic interaction patterns of the 162 mitochondrial DG (estimated TP rate = 85.8%). If diseases are linked to a genomic interval, the predicted DG can be prioritized from a larger list of functional candidates for mutational screen in affected individuals (**Table 3**). For example, the optic atrophy 2 (OPA2) linkage interval contains seven mitochondrial genes that include three known DG of which HSD17B10 is associated with optic atrophy [48]–[50], and three predicted DG of which two genes (NDUFB11, TIMM17B) interact with mitochondrial DG causing optic atrophy. Our phenome knowledgebase (www.mitophenome.org) can also be applied to investigate disorders through gene network association, in particular common conditions that are caused by single gene defects in a subset of patients [51]. For example, a search for Parkinson disease returns 12 mitochondrial DG with interactions to 24 predicted DG (e.g. CCS, MECR, PRKAR2B). Similarly, seizures and mental retardation, a common combination of mitochondrial features, is caused by 59 DG that interact with 124 predicted DG. With the decreasing cost of DNA sequencing, high-throughput screens linking phenotypes with genotypes will further increase the accuracy of gene-feature associations. To this end, easy navigation between clinical phenotype and gene information promises to aid in the recognition and diagnosis of mitochondrial disorders.

## Methods

### Mitochondrial disease genes

We identified 174 disease genes that encode proteins targeted to mitochondria (**Table S1**). While most gene defects are inherited as Mendelian traits; ACSL6, BAX, BCL2, ME2, MTHFD1, PARL, PHB, UCP1, and UCP2 are disease susceptibility alleles; DLST, OGDH, and PCK2 are disease-associated protein deficiencies; and HTRA2, MTCP1, SLC25A16, and WWOX cause disorders of unknown inheritance patterns. **Table S1** has also the annotations and PMID references (col. I) for intracellular protein localizations and the 39 genes encoding components of nine mitochondrial protein complexes and pathways (col. J). Additional mitochondrial genes (**Table S6**) were identified through integration of two studies [5],[36] that had combined predicted 1,200 mitochondrial genes.

### Phenotypic feature annotation

For each of the 174 disease genes, we identified individual studies and case reports describing a gene defect or deficiency and associated phenotype information. Manual extraction and annotation probably results in more specific and comprehensive data, with far fewer false-positives than automated alternatives including natural language processing and high-throughput screening [23]. Annotation of 1,636 full-text articles identified 461 phenotypic features that included the definition of specific clinical terms for each feature. Features were defined narrowly enough so that clinical diagnoses mapped to a single feature. This process was essential as phenotype descriptions were not consistent and often varied between different studies (see **Table S3**, col. E). To define features, we utilized standardized descriptors in the Medical Subject Headings (MeSH) database under the “Diseases” (coded under C), and the “Psychiatry and Psychology” (coded under F) branches of the MeSH hierarchy used by the National Library of Medicine (NLM). Individual matching of features with MeSH descriptors revealed their positions in the hierarchical MeSH ontology, together with the parent descriptor, the directly related hypernym feature. For this analysis, we assigned each feature to only one position in MeSH (e.g. diabetes mellitus has MeSH positions C18.452.394.750 and C19.246; we chose the first category). We only added hypernyms to the ontology that were directly related to at least two features. Some features were added manually because they had no clear match with MeSH descriptors. By integrating our 461 features with their 41 hypernyms, we identified 502 features that are hierarchically classified within the mitochondrial phenotype ontology (**Table S2**).

### Gene-feature pair annotation

We used 1,636 full-text articles to manually annotate 6,361 gene-feature pairs, each of which was created from at least one original PubMed article with a unique identifier (PMID). On average, we identified 2.77 PMID per gene-feature pair, and 9.87 PMID per gene. Further, we assigned each gene-feature pair to a unique OMIM disease record (e.g. 277900 for Wilson disease), which described the disorder and was referenced in many articles. We computationally integrated the 6,361 gene-feature pairs with our phenotype ontology, resulting in 10,202 gene-feature pairs (**Table S3**). This integration also assigned the PMID of each gene-feature pair to its directly related hypernym gene-feature pair. PMID assigned through ontology integration are labeled “#” (col. G). Because gene-feature pairs may be associated with more than one OMIM disease record, we consolidated the 10,202 gene-feature pairs (and their PMID) into 9,407 unique pairs. For example, the gene POLG is associated with ophthalmoplegia and three OMIM disease records (157640, 258450, 607459).

### Quantitative phenotypic associations (QPA)

The association ratio for each gene-feature pair is the fraction of PMID reporting a specific feature for a specific gene out of the total number of PMID annotated for that gene. The feature Fi specific association ratio for gene pair A–B (rFi) was calculated as:
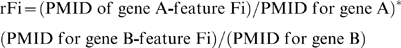
We considered the important weight wi of feature Fi for gene pair A–B as:
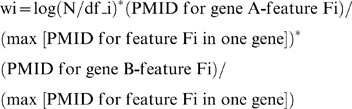
where N is the total number of genes (174) and df_i is the document frequency of feature Fi in all genes, which is related to the Inverse Document Frequency (IDF). IDF reflects the hypothesis that the value of a feature varies inversely with the number of genes in which it occurs [16]. To adjust for different PMID counts for a feature in different genes, we set a denominator as the maximum number of PMID in one gene associated with this feature. QPA integrated the association ratios (ri) in all features Fi (i = 1, 2, 3, …, I) for gene pair A–B through weights of wi_max which was calculated as:
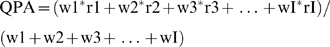



We identified feature Fi specific association ratios for 514,978 gene pairs (self-pairs removed), and computed QPA for 27,938 gene pairs with numerical values between 0.00177 and 1 (**Table S4**). An example of calculating QPA for specific disease genes is given under **Text S1**. In a related analysis, we studied associations of genes and features after removing eleven features that describe biochemical measurements of protein complexes (e.g. AKDH-deficiency, RCC1-deficiency). Removing these features resulted in 9,298 gene-feature pairs. We identified high correlations for QPA using the 9,298 versus all 9,407 gene-feature pairs, respectively (Kendall Cor = 0.9899, Spearman Cor = 0.9981, and Pearson Cor = 0.9978). While biochemical measurements support the diagnosis of many mitochondrial diseases, these features may not be essential for QPA predictions.

### Correlation of QPA and gene functional interactions

We extracted 2,010 disease gene pairs (140 genes) from a recent study that had integrated microarray co-expression, human and orthologous protein-protein interactions, and Gene Ontology (GO) similarities into Likelihood Ratios (LR) for gene functional interactions [34]. LR ranged from 1.1 to 2,374,581 and we used a linear transformation for LR to fall into a range between 0 and 1:
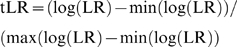



Overlapping all disease gene pairs with predictions for both LR and QPA revealed 1,928 pairs (**Table S4**). Two-sided tests of uniform distribution of LR and QPA for these pairs revealed non-uniform distribution with p values of 2.2e-16 (significance level: 0.05). Rank correlation of LR and QPA for the 1,928 gene pairs showed positive association with significant confidence: Kendall (Cor = 0.077; p = 4.67e-7), Spearman (Cor = 0.115; p = 3.72e-7), and Pearson (Cor = 0.145; p = 1.59e-10). Bivariate analysis and Lowess plots further confirmed this finding (**Figure S1**). In addition, we applied Euclidian distance and hierarchical average linkage clustering (http://www.r-project.org) and identified six gene clusters with higher to lower association of LR and QPA (**Figure S2** and **Table S4**).

### Functional interactions of nuclear-encoded mitochondrial genes

Out of the 174 DG, we identified functional interactions for 162 DG to 4,577 CG from a recent study [34]. As for the 162 DG, we also recorded all genome-wide binary interactions for the 4,577 CG with a total of 1,949,132 interactions. All gene interactions are non-redundant and non-self-interacting. The interactions for the 4,577 CG included interactions to additional 5,358 genes that we labeled CG interactors (CGin). We assigned the following four attributes to the total 10,097 genes that included DG, CG and CGin (**Table S6**): i. 1,283 disease genes identified through OMIM [26]; ii. 1,032 human orthologs to mouse essential genes [33]; iii. 977 human orthologs to yeast essential genes [37]; and iv. 863 nuclear-encoded mitochondrial genes through integrative analysis of two studies [5],[36]. We then computed for each DG (162) and CG (4,577) the total number of interactions (i[all-genes]), as well as all interactions to genes with the assigned attributes (i–iv). **Table S5** lists all genome-wide interactions for the 4,739 genes (col. C) and interactions to genes with the four attributes (col. D–G).

### Functional interactions of non-mitochondrial genes

The analysis of interactions of the non-mitochondrial CG (3551) and DG (493) showed the following results: i. the total number of interactions (i[all-genes]), which is recorded to all genes in the human genome, was higher for CG than for DG (p = 3.67e-3); ii. the relative number of DG interactions (i[disease-genes]/i[all-genes]), which was recorded to all known human DG, was higher for DG than for CG (p = 1.07e-21); and iii. the relative number of interactions to human orthologs of mouse (i[mouse-essential]/i[all-genes]) and yeast (i[yeast-essential]/i[all-genes]) essential genes [33],[37] was higher for the DG than the CG (mouse p = 3.77e-4; yeast p = 7.62e-7).

### Gene fraction analysis

We studied the number of interactions (degree) of genes in the different gene groups and the probability distribution of these interactions (degree distribution) over the whole network. For each gene group, we computed the degree distributions P(k) as the fraction of the number of genes that interact with k other genes, where the sum of fractions of a specific gene group is 1. Similar to the study by Goh et al. [33], we used log2k as the dependent variable in **Figure 3**. We calculated P(k) for interactions of each gene group to all human genes (**3A**), all human disease genes (**3B**) and all mitochondrial genes (**3C**) using data in **Table S5**. We then performed a fraction analysis by applying the linear regression model to the degree distributions of each gene group and attribute using the R statistical package (http://www.r-project.org) and calculated the values for the regression line slopes, their p-values and correlation coefficients (**Table S7**). We found that the measured trends described by the linear regression model are statistically significant for all gene groups with very small p-values (<10^−8^), which we obtained by testing the null hypothesis that the slope is zero. The negative regression slopes identified for all gene groups suggested a relatively higher portion of less-connected genes and a lower tendency to form a hub structure. We then ordered the gene groups using their slope values. The order was based on the hypothesis that as larger the absolute value of the negative regression slope, the higher the probability that lower-connected genes outnumber the higher-connected genes. This comparison showed that in the interactions to all genes (**3A**), DG mito showed relatively fewer interactions (−0.00142) than CG mito (−0.00072), and in the interactions to all disease genes (**3B**), DG mito showed relatively more interactions (−0.00612) than CG mito (−0.00776). Similar relationships were found in the comparison of the mitochondrial gene groups based on their correlation coefficients (**Table S7**). We concluded from these results that while mitochondrial disease genes displayed an overall smaller connectivity, they showed a tendency to interact with each other suggesting the formation of disease gene hubs in the periphery of the mitochondrial gene networks.

### Candidate genes for mitochondrial disorders

We applied the two supervised methods of discriminant analysis [43] and Bayesian network analysis [44],[45] to predict new mitochondrial DG. From **Table S5**, we selected 695 mitochondrial genes and their attributes of functional interactions and labeled the 162 DG as DG, the 495 CG as CG, and the 38 likely mitochondrial DG as CG to serve as controls. The linear discriminant covariance analysis was performed using the JMP statistical software with predictions results listed in **Table S8** (col. D–I). For the Bayesian network analysis (col. K–P), we first selected 100 typical DG out of the 162 DG, and 100 typical CG out of the 495 CG (col. K). The 200 genes were imported as training sets into a machine-learning algorithm and the Bayesian network package of this program was used to train the model by the method of cross validation [44]. We then imported the test set of all 695 mitochondrial genes in order to predict mitochondrial DG. The overlap of the two applied methods (col. Q) predicted 168 high-probability disease candidate genes out of the 495 non-disease CG.

### Author information

The accompanying mitochondrial phenome knowledgebase is available at http://www.mitophenome.org

## Supporting Information

Text S1 (0.03 MB DOC)

Figure S1 Lowess plot for the correlation of disease gene pairs predicted by LR and QPA.
(0.04 MB DOC)

Figure S2 Clustering of 1,928 disease gene-pairs with LR and QPA.
(0.08 MB DOC)

Table S1 174 nuclear-encoded mitochondrial disease genes
(0.09 MB XLS)

Table S2 502 phenotypic features in mitochondrial disease phenotype ontology
(0.06 MB XLS)

Table S3 9,407 annotated gene-feature associations
(1.90 MB XLS)

Table S4 27,938 QPA of disease gene pairs incl. 1,928 pairs with QPA and LR
(3.78 MB XLS)

Table S5 4,739 genes in functional network analysis and their 5 interaction attributes
(0.54 MB XLS)

Table S6 10,097 genes with LR interactions (DG, CG, and CG interactors)
(0.80 MB XLS)

Table S7 Gene fraction analysis results
(0.01 MB XLS)

Table S8 695 mitochondrial genes including newly predicted disease candidate genes.
(0.20 MB XLS)
