# Supplementary material for: Mapping Gene Associations in Human Mitochondria using Clinical Disease Phenotypes
Source: PLoS Comput Biol. 2009 Apr 24;5(4):e1000374. doi: 10.1371/journal.pcbi.1000374 (PMC2668170; doi:10.1371/journal.pcbi.1000374)
Supplement: Text S1 — (0.03 MB DOC) [file pcbi.1000374.s001.doc]

**Supporting information for calculating QPA between human disease genes**

Single case reports are arguably the most comprehensive and detailed descriptions of clinical disease phenotypes. Single cases are counted as 1 PMID in our analysis. If the original paper describing a medical case was available, only the PMID of this original paper was included and counted as 1 PMID. Because single case reports are sometimes not available or there number is not sufficient for analysis, multiple case reports were also included but their number is relatively small and evenly distributed across genes. In addition, to address potential limitations by using multiple case reports, the calculation of QPA takes into account the feature Fi specific association ratio for gene pairs instead of single genes, and the weight of a feature across all genes. In the following artificial and extreme example, we show the calculation of QPA for 2 PMID reporting 11 patients with defects in one gene, but where only 2 patients (1 in each PMID) are reported with the specific feature. For example, the first PMID reports the gene defect of SURF1 with the feature Leukoencephalopathy (MeSH ID C10.314.450) for 1 patient in a single case report. The second PMID describes 10 patients in a multiple case report, but only 1 out of 10 patients are reported with the feature of Leukoencephalopathy caused by defects in SURF1. If these two PMIDs would be the sole data used for analysis, then there are only two gene-feature association pairs: {SURF1, Leukoencephalopathy} and {SURF1, Leukoencephalopathy}. In this case, we are not calculating the QPA because there is only one gene (SURF1) involved in the feature Leukoencephalopathy. QPA is only calculated for genes associated with the same feature(s). However, it is very likely that additional three PMID’s exist, which report the gene-feature association pairs of {ABAT, Leukoencephalopathy}, {BCS1L, Leukoencephalopathy} and {SURF1, Ataxia}. Based on the total five PMID reports, we have the evidence of the following gene-feature association pairs: {SURF1, Leukoencephalopathy}, {SURF1, Leukoencephalopathy}, {ABAT, Leukoencephalopathy}, {BCS1L, Ataxia} and {SURF1, Ataxia}. Then, the feature Fi (F1 for Leukoencephalopathy; F2 for Ataxia) specific association ratio (rFi) for the gene pair A-B (SURF1-ABAT) is calculated as:

rF1 = (PMID for gene A-feature F1) / PMID for gene A) *

(PMID for gene B-feature F1) / (PMID for gene B) = 2/3 * 1/1 = 2/3

rF2 = (PMID for gene A-feature F2) / PMID for gene A) *

(PMID for gene B-feature F2) / (PMID for gene B) = 1/3 * 0/1 = 0

The important weight wi of feature Fi for gene pair A-B becomes:

w1_max = log(N/df_1) * (PMID for gene A-feature F1) / (max [PMID for feature F1 in one gene]) * (PMID for gene B-feature F1) / (max {PMID for feature F1 in one gene}) = log(3/2) * 2/2 * 1/2

w2_max = log(N/df_2) * (PMID for gene A-feature F2) / (max [PMID for feature F2 in one gene]) * (PMID for gene B-feature F2) / (max {PMID for feature F2 in one gene}) = log(3/2) * 1/1 * 0/1 = 0

where N is the total number of genes (3 genes in this example: SURF1, ABAT and BCS1L) and df_i is the document frequency of feature Fi in all genes, which is related to the Inverse Document Frequency (IDF). IDF reflects the hypothesis that the value of a feature varies inversely with the number of genes in which it occurs [Reference 16]. To adjust for different PMID counts for a feature in different genes, we set a denominator as the maximum number of PMID in one gene associated with this feature. QPA integrated the association ratios (ri) in the 2 features Fi (i=F1 and F2) for gene pair A-B through weights of wi_max, which was calculated as:

QPA = (w1*r1 + w2*r2) / (w1 + w2) = ({log(3/2) * 2/2 * 1/2 * 2/3} + {log(3/2) * 1/1 * 0/1 * 0}) / ({log(3/2) * 2/2 * 1/2} + {log(3/2) * 1/1 * 0/1} = 0.67

Thus, the QPA in this study has taken into account both information of the association strength in association ratios of rFi and the IDF in weights of wi_max. The statistical power in our study comes from the large amount of disease genes and medical cases we included.
