# Supplementary material for: Mapping Gene Associations in Human Mitochondria using Clinical Disease Phenotypes
Source: PLoS Comput Biol. 2009 Apr 24;5(4):e1000374. doi: 10.1371/journal.pcbi.1000374 (PMC2668170; doi:10.1371/journal.pcbi.1000374)

**Figure S1: Lowess plot for the correlation of disease gene pairs predicted by LR and QPA.** The plot shows the correlation of molecular interactions (transformed Likelihood ratios tLR; y axis) and quantitative phenotype associations (QPA, x axis) for 1,928 disease gene pairs predicted by both methods. While all 1,928 gene-pairs with both LR and QPA are used for the Lowess plot, the algorithm down weights outlier points with adjustment of LR ranges on the y axis.


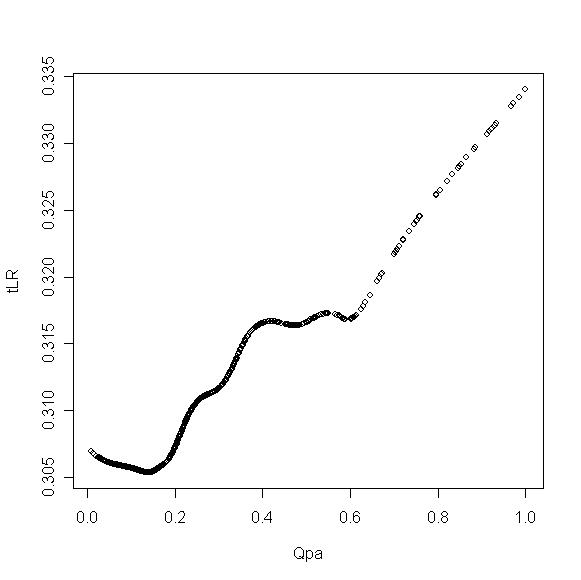

Supplement: Figure S1 — Lowess plot for the correlation of disease gene pairs predicted by LR and QPA. (0.04 MB DOC) [file pcbi.1000374.s002.doc]
