# Supplementary material for: Mapping Gene Associations in Human Mitochondria using Clinical Disease Phenotypes
Source: PLoS Comput Biol. 2009 Apr 24;5(4):e1000374. doi: 10.1371/journal.pcbi.1000374 (PMC2668170; doi:10.1371/journal.pcbi.1000374)
Supplement: Figure S2 — Clustering of 1,928 disease gene-pairs with LR and QPA. (0.08 MB DOC) [file pcbi.1000374.s003.doc]

**Figure S2: Clustering of 1,928 disease gene-pairs with LR and QPA.** Hierarchical average linkage clustering of the 1,928 disease gene-pairs predicted by both LR and QPA identified six groups of gene pairs with higher to lower associations of LR and QPA. Number of pairs in each group and maximum and minimum values for QPA and LR are shown in the table.


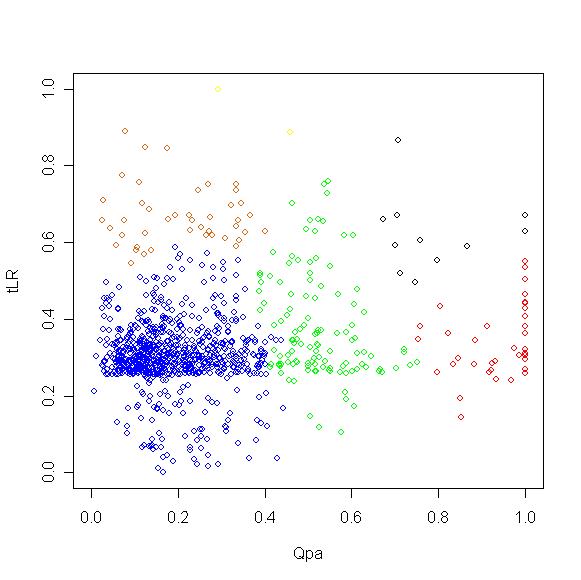


| **Cluster Groups –>** | **1 (blue)** | **2 (red)** | **3 (green)** | **4 (brown)** | **5 (black)** | **6 (yellow)** |
| --- | --- | --- | --- | --- | --- | --- |
| **Kendall tau** | 0.007108 | 0.271601 | -0.152212 | -0.023411 | -0.018518 | -1 |
| **p-value** | 0.680119 | 0.001530 | 0.000501 | 0.753756 | 0.908949 | 0.083264 |
| **QPA (max)** | 0.4435 | 1 | 0.75 | 0.4016 | 1 | 0.4585 |
| **QPA (min)** | 0.0083 | 0.7519 | 0.3878 | 0.0261 | 0.6731 | 0.2932 |
| **tLR (max)** | 0.5868 | 0.5516 | 0.7599 | 0.8887 | 0.8672 | 1 |
| **tLR (min)** | 0 | 0.1441 | 0.1052 | 0.5466 | 0.4966 | 0.8881 |
| **number of gene pairs** | 1504 | 74 | 238 | 86 | 22 | 4 |
